# Reduced levels of biomarkers of exposure in smokers switching to the Carbon-Heated Tobacco Product 1.0: a controlled, randomized, open-label 5-day exposure trial

**DOI:** 10.1038/s41598-020-76222-y

**Published:** 2020-11-05

**Authors:** Cam Tuan Tran, Marija Bosilkovska, Guillaume de La Bourdonnaye, Nicolas Blanc, Christelle Haziza

**Affiliations:** PMI Science & Innovation, Philip Morris Products S.A., Quai Jeanrenaud 5, 2000 Neuchâtel, Switzerland

**Keywords:** Biomarkers, Diseases

## Abstract

In addition to smoking cessation, for those who would otherwise continue to smoke, replacing cigarettes with less harmful alternatives can reduce the harms of smoking. Heating instead of burning tobacco reduces, or eliminates, the formation of harmful and potentially harmful constituents (HPHC) that are found in cigarette smoke. The Carbon-Heated Tobacco Product (CHTP), a heat-not-burn tobacco product, mimics the cigarette smoking ritual. This randomized, open-label, two-arm, parallel-group, short-term confinement study tested the hypothesis that the geometric means of the BoExp levels for subjects switching to CHTP 1.0 for 5 days are lower relative to those continuing to smoke cigarettes. Biomarkers of exposure (BoExp), including nicotine, urinary excretion of mutagenic constituents (Ames test), and cytochrome P450 (CYP) 1A2 activity, were measured in blood and/or 24-h urine samples during ad libitum product use. Nicotine exposure remained at similar levels in individuals using CHTP as in those continuing to smoke cigarettes. Switching to CHTP resulted in marked decreases in all other urinary BoExp (56–97%), carboxyhemoglobin (59%), urinary mutagenic constituents, and CYP1A2 activity compared with continued cigarette smoking. Our results provide evidence of decreased exposure to 15 selected HPHCs in smokers switching from cigarettes to exclusive CHTP use.

*Trial registration* ClinicalTrials.gov: NCT02503254; Date of first registration: 20/07/2015 https://www.clinicaltrials.gov/ct2/show/NCT02503254.

*Study protocol* Study protocol published at: https://www.clinicaltrials.gov/ProvidedDocs/54/NCT02503254/Prot_000.pdf.

## Introduction

Smoking cessation is the best approach for smokers to reduce the risk of diseases caused by smoking. However, despite the risks attributable to combusted tobacco, some smokers continue smoking, which demands another approach. Providing alternative less harmful products to adult smokers, who would otherwise continue using tobacco products, could reduce the deleterious health effects of combusted tobacco to the individual and to the population as a whole.

Various classes of nicotine delivery products that are potentially less harmful than cigarettes are currently offered to smokers as alternatives to cigarettes^1,2^. For tobacco-based products, heating tobacco (rather than burning it) prevents combustion and reduces the levels of harmful and potentially harmful constituents (HPHC) present in the generated aerosol in comparison to those found in cigarette smoke^3–6^.

From smokeless oral products and snus, to electronic cigarettes (e-cigarettes) and heated tobacco, the development of tobacco and nicotine-containing products as potential harmless alternatives to cigarettes, known as “potential reduced-exposure products”^7^, began decades ago. Since 2003, the prevalence of e-cigarettes (nicotine containing products) has increased^8^. E-cigarettes heat a liquid to generate a vapor, typically containing nicotine, which is inhaled.

Tobacco containing products include a variety of different products. In 1989, RJ Reynolds (RJR) released *Premier*, a product similar in size and appearance to a cigarette, hosting an aluminum canister containing alumina beads impregnated with tobacco extract and enabling vaporization through a carbon heat source^9^. *Eclipse*, a subsequent product also using a carbon-based heat source to heat a tobacco plug, was introduced in 1996 by RJR^10^, followed by *Revo*, a revamped version of *Eclipse*. In 1999, Philip Morris USA released *Accord*, a smoking system that electronically heated the tobacco when puffed, producing less smoke and no ash^11^.

Several electrically heated tobacco products are currently available on the market, such as Philip Morris International’s Tobacco Heating System (a candidate modified risk tobacco product [MRTP] sold under the brand name *IQOS*), Japan Tobacco’s *Ploom TECH*^12^, and British American Tobacco’s *Glo/iFuse*.

Tobacco products aiming at reducing the risk of harm and of smoking-related diseases compared with continued smoking are called MRTPs in the U.S.^13^ In this study we investigated a potential MRTP, which was test-marketed in the Dominican Republic in 2018, the Carbon-Heated Tobacco Product (CHTP) 1.0, developed by Philips Morris International. Despite a number of similarities with cigarettes in design and ritual, its technical design is fundamentally different and includes a carbon-based heat source, isolated from the tobacco plug, which contains specially processed tobacco. Once lit, the heat source provides energy to heat the tobacco plug to a well-defined temperature profile to avoid combustion. The heat source is isolated from the tobacco plug by a non-combustible, heat resistant, gas impermeable element, fixed to the rear portion of the heat source (Supplementary Figure 1). This element provides a physical separation between the heat source and tobacco plug, preventing heat source emissions from entering the aerosol generated from the tobacco plug^14^. The aerosol is composed primarily of water, glycerol (as humectant), nicotine, and significantly reduced amounts of HPHCs^15^.

A prototype of CHTP (version MD2-E7) was tested in a clinical study in 2009 (ClinicalTrials.gov, NCT00812279) in 112 adult smokers randomized to three groups: continuing to smoke cigarettes, switching to the CHTP, or smoking abstinence (SA). Subjects in the CHTP group had decreased levels from baseline (levels measured during the period after the enrollment but before allocation to the respective group by randomization) comparable to the SA group in all measured biomarkers of exposure (BoExp) to HPHCs (e.g., carboxyhemoglobin [COHb]) and lower urinary excretion of mutagenic constituents after 5 days relative to cigarette smoking^15^.

CHTP 1.0, based on the earlier prototypes, was designed to address consumer needs to deliver sufficient nicotine per puff and emulate the experience of cigarette smoking in terms of ritual while having the potential to present less risk of harm. Compared with the CHTP MD2-E7 prototype, changes were made to the design of the heat source and filter paper of CHTP 1.0 in order to yield higher levels of nicotine in the aerosol [0.50 mg and 1.30 mg under International Organization for Standardization (ISO) and Health Canada Intense (HCI) smoking regimes, respectively, compared with 0.40 mg and 0.68 mg, respectively, for MD2-E7]. In order to test the newly improved CHTP 1.0 in terms of performance and acceptance, the present study was designed to demonstrate a reduction in HPHC exposure in adult healthy smokers switching from their preferred brand of cigarettes to CHTP 1.0 for 5 days. In addition, product use behavior (e.g., puff rate and inhalation depth), amount of product use, nicotine uptake, urinary excretion of mutagenic constituents, and cytochrome P450 (CYP) 1A2 activity were assessed. The primary objective of the study consisted of demonstrating the reduction of exposure in four biomarkers of exposure (monohydroxybutenylmercapturic acid [MHBMA] a biomarker of exposure to 1,3-Butadiene, 3-hydroxypropylmercapturic acid [3-HPMA] a biomarker for Acrolein, S-phenylmercapturic acid [S-PMA] a biomarker for Benzene, and COHb, a biomarker for CO).

## Materials and methods

### Study design

This controlled, randomized, open-label, two-arm parallel group, single-center confinement study was conducted between July and September 2015 at the BioVirtus Research Site (Kajetany, Poland) and was registered at ClinicalTrials.gov (NCT02503254). The study was approved by the independent ethics committee of the Regional Medical Chamber of Physicians in Warsaw, Poland.

**Figure 1:**
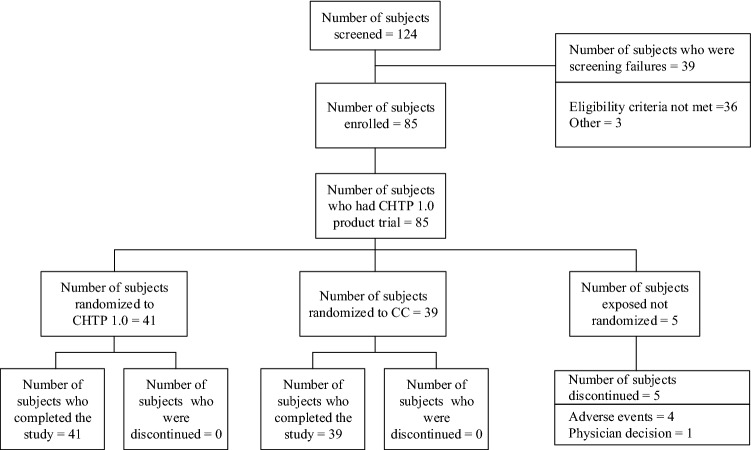
Subject disposition. *CC* cigarette, *CHTP* Carbon-Heated Tobacco Product.

A total of 85 eligible adult subjects were enrolled on Day − 3 (Admission) when CHTP was tested post-enrollment. Five subjects were discontinued (Fig. 1) before randomization. On Day − 1, 80 subjects were randomized by an interactive web and voice response system at a 1:1 ratio to either switching to CHTP 1.0 (CHTP group, n = 41) or to continue cigarette smoking (cigarette group, n = 39). Subjects were stratified for randomization by sex and self-reported average daily cigarette consumption in the six weeks prior to admission (10–19 cigarettes or > 19 cigarettes per day). From Days 1 to 5, ad libitum product use of the allocated product was allowed, with products dispensed upon subjects’ request and recorded by the site staff. After discharge on Day 6, subjects were followed up for 7 days for ongoing and new spontaneously reported adverse events (AE) or serious adverse events (SAE).

### Subjects

Subjects were recruited from the clinic’s database and through advertisements. Compensation was provided to subjects, as per IRB approval and according to a pre-defined payment schedule, irrespective of their actual tobacco product use. Male and female Caucasian adult smokers meeting the following criteria were eligible for this study: age ≥ 21 years; body mass index (BMI) 18.5–32.0 kg/m^2^; daily smoking habit of at least 10 non-menthol cigarettes of any brand, with a maximum yield of 1 mg nicotine per cigarette (maximum ISO criteria), for six weeks prior to admission; had smoked for at least three years prior to screening; not intending to quit smoking in the next three months; and healthy, as judged by the investigator using available clinical and laboratory parameters (medical history, physical examination, vital signs, standard hematology, clinical biochemistry and urinalysis parameters, spirometry, electrocardiogram, and chest X-ray). Pregnant or breastfeeding women were excluded, as well as women of childbearing potential, if they did not use an effective contraceptive method. After receiving a full explanation of the study, subjects signed informed consent forms prior to any assessments.

### Assessments

The revised version of the self-reported Fagerström Test for Nicotine Dependence (FTND) questionnaire^16^ was completed by subjects at the Screening Visit. BoExp to 15 different HPHCs measured in this study are presented in Supplementary Table 1. BoExp were assayed as described in a previous publication^17^. To assess urinary BoExp, measurements were performed on 24-h urine samples at baseline and throughout the 5-day exposure period. Creatinine was determined in the same samples, and its levels were used to adjust BoExp concentration values. COHb was measured in blood samples collected each evening. Selection of HPHCs was based on recommendations for lowering constituents in cigarette smoke, as defined by the World Health Organization (WHO)^18^ and the U.S. Food and Drug Administration^19^. Nearly all BoExp assessed in this study have elimination half-lives ≤ 24 h, except for total 4-(methylnitrosamino)-1-(3-pyridyl)-1-butanol (NNAL), with an estimated half-life of 10–45 days^20^. Therefore, except for total NNAL, 5 days of product use were enough to observe optimal decreases in BoExp levels.

CYP1A2 activity, which is involved in the activation of carcinogenic heterocyclic and aromatic amines and is induced by polycyclic aromatic hydrocarbons (PAH), was assessed in plasma by measuring paraxanthine (PX) and caffeine (CAF) concentrations and calculated as PX/CAF molar metabolic ratios. Blood samples for these measurements were collected approximately six hours (± 15 min) after drinking a cup of coffee (NESCAFÉ Gold, Nestlé, Germany) providing approximately 150 mg of CAF at baseline and Day 5^21^. At baseline and Day 5, the excretion of mutagenic constituents was measured in a 24-h urine sample using the Ames assay, with results expressed as the number of revertants per 24 h (REV/24 h).

Nicotine uptake was assessed by nicotine and cotinine evening concentrations in plasma and by 24-h urinary excretion of nicotine equivalents (NEQ) from samples collected at baseline and throughout the 5-day randomized period^22^.

Parameters to assess human puffing behavior (Supplementary Table 5) were measured on Day –2, Day 1, and Day 4 using the human puffing topography (HPT) SODIM device model SPA/M (SODIM Instrumentation, Fleury les Aubrais, France) described previously^15^. The sample holders for the HPT SODIM device were designed for compatibility with CHTP 1.0 sticks. During cigarette smoking, HPT was assessed only for cigarettes that were compatible with the mouthpiece of the HPT device (i.e., excluding slim cigarettes).

Safety, including abnormal laboratory findings and AE recording, was monitored throughout the study. AEs were assessed for their relationships to CHTP and cigarettes and whether they were expected.

### Tobacco products

The CHTP 1.0 tested in this study [0.50 mg nicotine yield (under ISO smoking regimen) and 1.30 mg nicotine yield (under HCI smoking regimen), respectively, by heating tobacco within a well-defined temperature profile to avoid combustion] was a non-menthol tobacco stick with a shape, form, and use similar to a cigarette (Supplementary Figure 1). It is a single-use product (disposed of after one use experience). Each product use experience has a duration of approximately 5 min, until the taste fades away and the experience concludes. Reference and baseline product was a commercially available non-menthol cigarette of the subject’s preference, with ISO nicotine yield of up to 1 mg. Subjects were asked to purchase their own preferred single-brand of non-menthol CC prior to enrolment. Each subject bought his/her anticipated amount of single-brand CC for a total of 9 days plus 2 extra packs. As CHTP 1.0 was not commercialized at the time of the study, CHTP 1.0 tobacco sticks were provided to subjects randomized to the CHTP arm.

### Data analysis

The primary objective of the study consisted of demonstrating the reduction of exposure in four biomarkers of exposure (monohydroxybutenylmercapturic acid [MHBMA], 3-hydroxypropylmercapturic acid [3-HPMA], S-phenylmercapturic acid [S-PMA], and COHb). The hypothesis to be tested was that levels of each of the four biomarkers of exposure were reduced by more than 50% in subjects switching to CHTP 1.0.

Based on the results from a previous study on the CHTP MD2-E7 prototype^15^, a sample size of 40 subjects per group for a two-arm comparison was sufficient to demonstrate, with 80% power, a 50% reduction in each co-primary BoExp using a one-sided test with 2.5% type I error probability. All remaining BoExp were assessed as secondary objectives.

BoExp were analyzed in all randomized subjects who used the allocated product at least once after randomization and with at least one valid BoExp measure after product use. BoExp and HPT parameters were expressed on a log scale. Analyses of covariance (ANCOVA) between study groups were conducted for Day 5 values of each parameter, with adjustments for sex, average cigarette consumption over the six weeks prior to admission, and baseline values. Least square mean reductions for CHTP vs. cigarette groups and their confidence intervals (CI) were calculated from the ANCOVA models. All statistical analyses were performed using Statistical Analysis Software (SAS) version 9.2 (SAS Inc., Cary, NC, USA).

### Ethical approval

The study was approved by the independent ethics committee of the Regional Medical Chamber of Physicians in Warsaw, Poland. All procedures performed in studies involving human participants were in accordance with the ethical standards of the institutional and/or national research committee and with the 1964 Helsinki declaration and its later amendments or comparable ethical standards.

### Informed consent

Informed consent was obtained from all individual participants included in the study.

## Results

Of 124 screened subjects, 85 were enrolled in the study. Five subjects were discontinued prior to randomization: four with AEs or SAEs (two hypertriglyceridemia, one leukocyturia, and one concussion), and one because of blood sampling difficulties, as decided by the Principal Investigator. Eighty subjects were randomly assigned to the CHTP (n = 41) or cigarette (n = 39) groups, and all completed the study (Fig. 1).

No substantial differences in sex, age, BMI, FTND score, or daily cigarette consumption were observed between the study groups at baseline (Table 1).

**Table 1:** Demographic characteristics by study group at baseline.

Characteristics	CHTP (n = 41)	Cigarettes (n = 39)
Male n (%)	20 (48.8)	19 (48.7)
Age, mean ± SD	34.1 ± 10.45	32.7 ± 10.97
BMI normal weight n (%)	20 (48.8)	21 (53.8)
BMI, mean ± SD	25.6 ± 3.3	25.1 ± 3.1
**Daily cigarette consumption n (%)**		
10–19/day	21 (51.2)	19 (48.7)
> 19/day	20 (48.8)	20 (51.3)
**ISO tar yield n (%)**		
1–5 mg	7 (17.1)	6 (15.4)
6–8 mg	26 (63.4)	29 (74.4)
9–10 mg	8 (19.5)	4 (10.3)
**ISO nicotine yield n (%)**		
≤ 0.6 mg	32 (78.0)	34 (87.2)
> 0.6–1 mg	9 (22.0)	5 (12.8)
FTND score, mean (SD)	5.4 (1.78)	5.8 (2.00)

*CHTP*, Carbon-Heated Tobacco Product 1.0, *SD* standard deviation, *BMI* body mass index, *FTND* Fagerström Test for Nicotine Dependence, *ISO* International Organization for Standardization.

### Biomarkers of exposure

On Day 5, COHb, MHBMA, 3-HPMA, and S-PMA levels in the CHTP group were decreased, with reductions ranging from 50.4 to 84.2%, when compared to Baseline. In contrast, COHb, MHBMA, 3-HPMA, and S-PMA levels in the cigarette group were increased on Day 5 ranging from 7.6 to 32.2%, when compared to Baseline (Table 2). When compared to Baseline, other BoExp levels in the CHTP group were reduced on Day 5 ranging from 52.7 to 95.8%, whereas they were increased in the cigarette group ranging from 4.7 to 30.3%.

**Table 2:** Biomarkers of exposure levels at baseline and Day 5.

Biomarker	CHTP (n = 41) Estimate (95% CI)	Cigarettes (n = 39) Estimate (95% CI)
**COHb (%)**		
Baseline	5.8 (5.3; 6.3)	5.6 (5.1; 6.2)
Day 5	2.7 (2.2; 3.2)	6.4 (5.7; 7.1)
% change	− 50.4 (− 58.8; − 41.9)	7.6 (− 0.1; 15.2)
**MHBMA (pg/mg creatinine)**		
Baseline	1635.31 (1245.24; 2147.55)	1466.37 (1048.61; 2050.56)
Day 5	339.73 (301.82; 382.42)	1840.61 (1275.38; 2656.32)
% change	− 72.6 (− 80.0; − 65.3)	32.2 (18.5; 45.9)
**3-HPMA (ng/mg creatinine)**		
Baseline	1086.68 (928.83; 1271.35)	947.83 (817.32; 1099.18)
Day 5	494.70 (417.53; 586.12)	1187.97 (1026.63; 1374.65)
% change	− 53.0 (− 56.9; − 49.2)	28.1 (19.6; 36.6)
**S-PMA (pg/mg creatinine)**		
Baseline	2432.70 (1919.90; 3082.47)	2301.95 (1732.67; 3058.27)
Day 5	361.48 (289.26; 451.74)	2898.46 (2172.62; 3866.79)
% change	− 84.2 (− 85.9; − 82.6)	28.9 (19.9; 37.8)
**Total 1-OHP (pg/mg creatinine)**		
Baseline	242.85 (210.44; 280.25)	194.25 (166.36; 226.82)
Day 5	106.33 (93.18; 121.33)	199.12 (171.73; 230.88)
% change	− 55.3 (− 58.3; − 52.2)	4.7 (− 2.5; 12.0)
**4-ABP (pg/mg creatinine)**		
Baseline	16.76 (14.50; 19.37)	14.56 (12.61; 16.82)
Day 5	3.71 (3.28; 4.18)	15.91 (13.79; 18.34)
% change	− 76.5 (− 79.2; − 73.8)	11.0 (4.5; 17. 6)
**1-NA (pg/mg creatinine)**		
Baseline	93.14 (81.79; 106.06)	90.67 (78.32; 104.96)
Day 5	3.44 (2.82; 4.20)	115.76 (100.49; 133.35)
% change	− 95.8 (− 96.5; − 95.0)	30.3 (21.9; 38.6)
**2-NA (pg/mg creatinine)**		
Baseline	27.45 (24.11; 31.26)	25.07 (21.53; 29.20)
Day 5	3.15 (2.70; 3.69)	29.37 (25.36; 34.02)
% change	− 87.7 (− 89.1; − 86.4)	18.8 (12.3; 25.4)
**o-tol (pg/mg creatinine)**		
Baseline	152.30 (137.11; 169.16)	149.95 (126.73; 177.42)
Day 5	49.26 (43.59; 55.67)	175.40 (156.97; 195.99)
% change	− 65.5 (− 69.8; − 61.2)	24.4 (9.3; 39.4)
**CEMA (ng/mg creatinine)**		
Baseline	130.08 (107.27; 157.74)	126.88 (107.86; 149.25)
Day 5	20.78 (16.66; 25.92)	143.18 (122.59; 167.22)
% change	− 83.3 (− 84.9; − 81.7)	15.3 (7.7; 22.8)
**HEMA (pg/mg creatinine)**		
Baseline	3123.34 (2305.75; 4230.81)	2876.32 (2307.89; 3584.76)
Day 5	1146.84 (913.06; 1440.48)	3094.70 (2477.69; 3865.35)
% change	− 58.3 (− 66.1; − 50.6)	11.8 (1.6; 22.1)
**HMPMA (ng/mg creatinine)**		
Baseline	481.38 (428.98; 540.17)	451.09 (394.98; 515.17)
Day 5	125.75 (110.46; 143.16)	484.18 (420.56; 557.41)
% change	− 72.9 (− 75.2; − 70.6)	10.1 (1.9; 18.4)
**Total NNAL (pg/mg creatinine)**		
Baseline	162.41 (129.89; 203.06)	106.86 (83.69; 136.43)
Day 5	74.70 (59.90; 93.17)	117.82 (91.61; 151.53)
% change	− 52.7 (− 56.3; − 49.1)	12.5 (5.1; 19.9)
**3-OH-B[a]P (fg/mg creatinine)**		
Baseline	145.72 (119.17; 178.19)	107.10 (87.22; 131.50)
Day 5	33.23 (27.37; 40.35)	116.77 (95.09; 143.40)
% change	− 72.4 (− 79.9; − 65.0)	12.5 (3.3; 21.7)
**Total NNN (pg/mg creatinine)**		
Baseline	7.04 (5.19; 9.56)	4.99 (3.91; 6.36)
Day 5	2.13 (1.73; 2.63)	6.12 (4.87; 7.70)
% change	− 59.2 (− 68.6; − 49.8)	28.6 (16.3; 40. 8)

Biomarkers of exposure from 24-h urine creatinine-adjusted samples (COHb measured in blood). Baseline and Day 5 values presented as geometric mean (95% CI). % change refers to mean percent change from baseline to Day 5. Acronyms of biomarkers are provided in Supplementary Table 1.

*CHTP* Carbon-Heated Tobacco Product 1.0., *CI* confidence interval.

**Figure 2:**
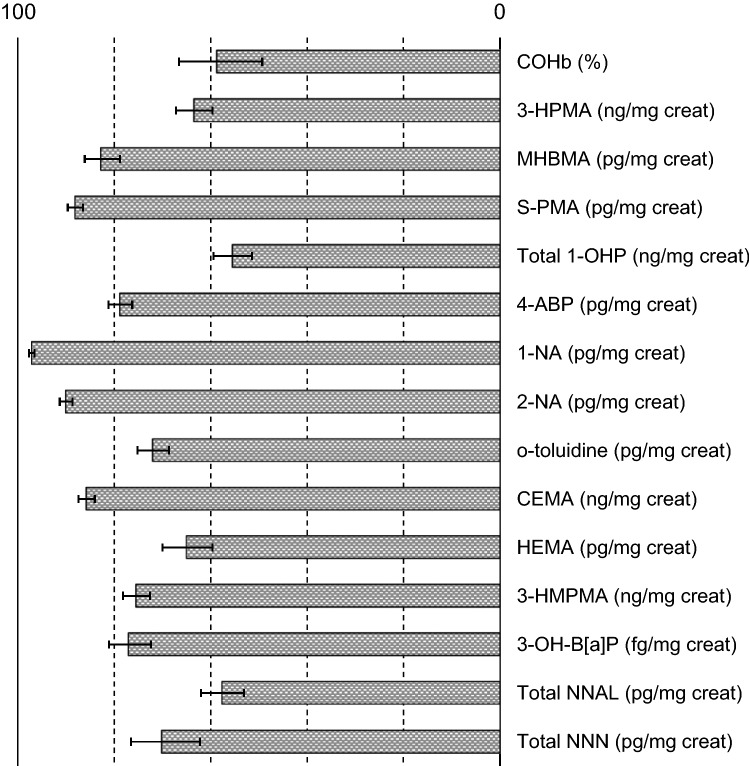
Biomarkers of exposure level reductions (%) on Day 5, CHTP relative to cigarettes. Values are geometric least square mean ratios and 95% confidence intervals. Acronyms of biomarkers of exposure are provided in Supplementary Table 1. *CHTP 1.0* Carbon-Heated Tobacco Product 1.0.

On Day 5, COHb, MHBMA, 3-HPMA, and S-PMA levels in the CHTP group were decreased, with reductions ranging from 58.8 to 88.1% compared with the cigarette group. Reductions in the other BoExp levels in the CHTP group ranged from 55.6 to 97.1% compared with the cigarette group (Fig. 2 and Supplementary Table 2).

### Biological activities

At baseline, the mean (standard deviation [SD]) values of revertants in the Ames mutagenicity test were 25,780 (20,618) REV/24 h in the CHTP group and 18,882 (14,538) REV/24 h in the cigarette group, respectively. On Day 5, these values decreased relative to baseline values in CHTP users to 6044 (5166) REV/24 h, while those in the cigarette group increased to 24,780 (14,512) REV/24 h.

At baseline, the mean SD CYP1A2 activity was 93.8% (30.4) in the CHTP group and 91.8% (32.5) in the cigarette group. On Day 5, mean CYP1A2 activity in CHTP users decreased by 22.0% (17.7) and increased by 4.9% (16.1) in the cigarette group. This reflected a difference in CYP1A2 activity of 24.3% (95% CI 30.2, 18.4) in the CHTP group when compared with the cigarette group.

### Product use and nicotine exposure

Supplementary Table 3 summarizes daily product consumption throughout the study. Baseline average daily cigarette consumption was 18 and 17 per day for the CHTP and cigarette groups, respectively. The mean number of CHTP sticks consumed daily in the CHTP group was slightly lower than at baseline on Day 1 but was higher than baseline from Day 2 through Day 5. In the cigarette group, the mean number of cigarettes consumed daily was lower than baseline from Day 1 through Day 4 and higher on Day 5. In both groups, the quantity of CHTP or cigarettes consumed on Day 5 was higher than baseline, though this increase was greater in the CHTP group.

Nicotine uptake during the study is summarized in Supplementary Table 4. NEQ values and plasma cotinine and nicotine levels on Day 5 were higher than at baseline, with comparable values in the two groups.

### Human puffing topography

HPT parameters were stable from baseline through Day 4 in the cigarette group. In the CHTP group, there were significant changes in HPT parameters on Day 1, which were even more pronounced on Day 4 (Supplementary Table 5). Smokers who switched to CHTP puffed more intensively, as indicated by the increased puff numbers, durations, and volumes. After 4 days of use, switching to CHTP led to a 70% increase in total puff volume compared with puff volume drawn by smokers when smoking their cigarette.

### Safety

Thirty-one of 41 subjects (75.6%) in the CHTP group and 20 of 39 subjects (51.3%) in the cigarette group reported at least one AE. The number of AEs and number of subjects reporting AEs were higher in the CHTP group. The most common AEs were cough (reported only in the CHTP group) and headache (reported in both groups). No SAEs were reported by any randomized subject, and no subject was discontinued because of an AE or SAE. All AEs were either mild or moderate in severity. The most frequent AE was headache, reported by 19 subjects (46.3%) in the CHTP group, 9 subjects (23.1%) in the cigarette group, and 3 (60%) exposed to CHTP during product testing but not randomized. A total of 13 subjects (31.7%) reported cough, which was assessed as related to CHTP in 12 subjects (29.3%), occurring at the beginning of the CHTP use period. All other AEs were reported in < 5% of enrolled subjects. AEs are presented in detail in Supplementary Table 6.

## Discussion

We investigated changes in BoExp in adult smokers in a controlled clinical setting. Randomization and strict monitoring of subjects, together with full control of product distribution and product use in confinement aiming to avoid dual use of cigarettes and CHTP, allowed for an optimal evaluation of the effects that can be achieved by switching from cigarettes to CHTP use exclusively for 5 days.

### BoExp to selected cigarette smoke HPHCs

COHb levels in smokers correlate with the number of cigarettes smoked per day^23,24^. Typical COHb levels are 4–8% in smokers and 0.8–1.5% in non-smokers^25^. In our study, COHb values at baseline were 5.6–5.8%, in the expected range for smokers, and decreased to 2.7% (50.4% decrease from baseline) after 5 days of CHTP use. This was consistent with the 60% decrease from baseline observed in a previous study, testing the CHTP MD2-E7 prototype^15^, as well as results obtained with *IQOS* and *Glo*, measuring exposure to CO in users (decrease from 49 to 76% measured by COHb or exhaled CO)^3–6,17^. The decrease in carbon monoxide exposure observed when switching from cigarettes to CHTP 1.0 was also consistent with the reductions from 49 to 79%^3–5,17^ reported in other studies in the literature upon 5 days of SA.

In non-smoking populations with coronary artery disease, COHb levels not exceeding 2.4–2.5% are recommended to avoid hypoxic effects^26^. After 5 days, the COHb level in the CHTP group was slightly above (2.7%) the recommended values of 2.4–2.5% from WHO for smokers at risk for cardiovascular diseases^18^. The slightly higher levels observed are unlikely an effect of CHTP but rather an overestimate bias at low COHb concentrations of ≤ 2.5% when using spectrophotometry-based methods, as shown by Mahoney et al. in a study comparing blood COHb measurement results by spectrophotometers versus the reference method, namely the gas-chromatographic method, for accurate COHb determinations^27^. A study conducted in Japan^5^ showed a 51% decrease in COHb levels after 5 days of *IQOS* use (47% compared with subjects continuing smoking) and a 53% decrease upon smoking cessation, ending with average COHb levels of 2.4% in the *IQOS* and SA groups.

Urinary excretion of MHBMA increased 2- to 40-fold in smokers compared with non-smokers, with a tenfold average difference^23^. Studies of SA for 3–8 days showed MHBMA levels 6- to 30-fold lower than baseline values, with an average reduction of approximately tenfold^28^. In our study, urinary MHBMA excretion was decreased by 83% (approximately sixfold) in the CHTP group compared with the cigarette group^17^. Our findings are also in agreement with results obtained with the CHTP MD2-E7 prototype^15^ and with a similar study conducted with *IQOS*^3–5,17^.

Levels of urinary 3-HPMA among smokers and non-smokers can overlap, but smokers usually have higher levels than non-smokers, with ethnic differences observed^29^. In several studies, after 5–8 days of SA, there were 1.5- to 8-fold decreases in urinary 3-HPMA excretion^24^. Acrolein, the corresponding HPHC, is ubiquitous in the environment, being naturally present in food and formed during food preparation and fuel combustion. Acrolein is also formed from tobacco at 50–500 °C^30^, within CHTP operating temperatures. In our study, the 64% decrease in 3-HPMA levels in the CHTP group compared with the cigarette group was in line with previous findings from the CHTP MD2-E7 prototype^15^ and *IQOS*^3–5,17^.

Levels of S-PMA are higher in smokers than in non-smokers^23,31^. After 3–8 days of SA, urinary S-PMA levels decreased from 2- to 20-fold^32,33^. In our study, the average reduction in urinary excretion of S-PMA in the CHTP group after 5 days was 88% (a nearly tenfold decrease) compared with the cigarette group. These results were comparable with previous findings with heat-not-burn tobacco products^3–5,15,17^.

Levels of all the other BoExp decreased in the CHTP group from 56% (total 1-hydroxypyrene) to 97% (1-aminonaphthalene) compared with the cigarette group. These effects were comparable with heat-not-burn tobacco products in other studies^3–5,15,17^.

Tobacco-specific nitrosamines (TSNA), mainly 4-(methylnitrosamino)-1-(3-pyridyl)-1-butanone (NNK) and *N*-nitrosonornicotine (NNN), have received particular attention because of their associated cancer risks. TSNAs occur widely in tobacco and are formed by nitrosation of nicotine and other tobacco alkaloids during tobacco curing^34^. The nature of the curing process influences the quality of the processed tobacco and its TSNA content. NNK and NNN are transferred to cigarette smoke upon vaporization at distillation and pyrolysis temperatures^35,36^. Exposure to NNK and to NNN was assessed in our study through their respective BoExp, total NNAL and total NNN. Compared with the cigarette group, total NNAL and total NNN decreased by 57% and 70%, respectively, in the CHTP group, comparable with previously reported effects of SA and heat-not-burn tobacco products^3–6,17^. Because the elimination half-life of total NNAL is several weeks^20,37^, 5 days is likely not long enough to observe an optimal reduction. Therefore, one to three months are potentially needed to achieve steady-state levels indicating decreased exposure.

### Biological activities

Combustible cigarette smoke contains many mutagenic and carcinogenic compounds, including nitroso-compounds, PAHs, and heterocyclic amines, usually undergoing urinary or fecal excretion. Thus, the urinary level of mutagens reflects both exposure doses and metabolic states of these carcinogens and mutagens^38^. Switching to the CHTP MD2-E7 prototype^15^ led to a decrease in urinary mutagens of 90% relative to baseline compared with 77% for CHTP 1.0 in our study. However, the number of revertants on Day 5 was similar in both studies, 6044 REV/24 h and 6600 REV/24 h with CHTP 1.0 and CHTP MD2-E7, respectively, while baseline values were more than threefold higher in the previous study compared with the present study. Overall, the decreased urine mutagenicity values in both studies indicate a marked reduction in exposure to potential mutagens^24^. Similar effects on urine mutagenicity were observed with *IQOS*^15^.

The CYP1A2 enzyme is involved in the metabolism of many foreign substances as well as the activation of carcinogenic heterocyclic and aromatic amines. The CYP1A2 phenotype is highly variable, in part because the enzyme is inhibited or induced by foreign substances, environmental compounds, diet, or lifestyle^21^. CYP1A2 expression is induced to a large extent by PAHs, which are found in cigarette smoke; thus, heavy smokers had 1.72 times higher CYP1A2 activity than non-smokers. Smoking cessation for one to two weeks resulted in a 36% decrease in CYP1A2 activity^21^. In our study, CYP1A2 activity in CHTP users was decreased by 22% from baseline in the CHTP group compared with the cigarette group after 5 days. Similar decreases in CYP1A2 levels were reported with IQOS, with CYP1A2 activity decreasing on Day 5 by 21–33%, which is similar to levels for SA^17^.

### Product use, HPT, and nicotine exposure

Nicotine uptake (i.e., the amount of absorbed nicotine) results from complex interactions between the amount of nicotine delivered from the CHTP tobacco plug, puffing behavior of the user (such as puff volume, intensity, and duration), intensity of inhalation, the way the users inhale the aerosol, nicotine intrinsic distribution within the body (the amount of nicotine reaching the lung, thus driving the rate and amount of nicotine absorption), and daily number of CHTP units consumed. It has been described that one of the mechanisms by which smokers titrate desired doses of nicotine when switching to new cigarette alternatives is to modify their puffing behavior (e.g., by taking higher puff volumes or to increase their daily product use) during a “learning period” that can take several weeks^39,40^.

The daily product use was increased in both groups after 5 days compared with baseline consumption but was higher in the CHTP group than in the cigarette group, while nicotine exposure was comparable between both groups. These results indicate that subjects in the CHTP group were able to self-titrate their nicotine uptake to desired levels, despite the short period of adaptation to the novel alternative tobacco product.

The substantial change in puffing behavior when using CHTP is likely to be the underlying way for titration and adjustment to CHTP use.

Substantial adaptation in puffing behavior was needed with the CHTP MD2-E7 prototype to achieve desired levels of nicotine exposure, requiring increased numbers of puffs, average puff volumes, and average puff durations. This led to 190%–276% increases in total puff volume (reaching approximately 2000 mL per CHTP MD2-E7 stick) compared with cigarette smoking^15^. For cigarettes, it has been shown that the puff volume and total volume are the two parameters with the greatest effect on delivery of HPHCs, such as carbonyls, to smokers during smoking, whereas the puff duration and number of puffs have minimal effects on toxicant delivery^41^. Our study showed an average 70% increase in total puff volume per CHTP stick, compared with cigarette smoking, which reflected how subjects adapted to this new product during an initial learning period. Compared to the previous product, there were changes to the design of the heat source and the filter paper. However, these changes gave no reason to suggest a change in puffing behavior.

Contrary to what has been described in the literature for cigarettes, the increase in total puff volume per CHTP stick in our study did not lead to increased delivery of HPHCs but to significantly decreased levels of selected BoExp to HPHCs compared with cigarettes. The combustion of tobacco during cigarette smoking exposes smokers to more than 6,000 harmful HPHCs. The generation profiles of known toxic compounds in tobacco smoke vary according to the pyrolysis temperature (300–1000 °C) and the pyrolysis atmosphere (in nitrogen and air). Data published by Torikai, K et al. clearly show increases of toxicant generation with increasing temperatures^30^. In contrast, the tobacco plug in CHTP is heated within a well-defined temperature profile, to avoid combustion, which results in a substantial decrease in the generation of HPHCs in the aerosol^42–44^.

### Safety

Coughing and headache were observed in the CHTP group more frequently than in the cigarette group, and we attribute the higher frequency of coughing to the decreased HPHC exposure. Several studies have demonstrated that prolonged exposure to cigarette smoke suppresses the cough reflex, whereas smoking cessation, eliminating exposure to smoke constituents, increases the cough reflex within two weeks, leading to a higher cough frequency^45^. A transient increase in respiratory symptoms, increased cough, and sputum production during the first 2 days of smoking cessation has been discussed in the literature^45–49^. The proposed mechanism is a rapid re-sensitization of cough reflex receptors upon smoking cessation^50^.

Subjects in both groups reported headaches, with the CHTP group reporting twice as many as the cigarette group. Although headaches have also been observed more frequently in smokers who use smoking cessation pharmacological treatments, such as nicotine replacement therapy, the underlying mechanism remains unclarified^51^.

### Study limitations and strengths

The study duration was short; therefore, some subjects may not have fully adapted their puffing behavior to the CHTP. The study conditions and procedures were tightly controlled, preventing product use behaviors more comparable to real-world conditions. Although subjects could use the products ad libitum during the study, they were required to ask for each cigarette or CHTP stick, which may have affected their consumption behavior. Daily consumption increased from baseline to the end of the exposure period in both groups, consistent with findings in other studies that laboratory confinement conditions promoted higher consumption of tobacco products. The greatest decreases in HPHC exposure are observed during sustained SA. Other than NNK and NNN, all other HPHCs examined could be confounded with other potential sources, including food and occupational or environmental (e.g., air pollution) exposures. Because there was no SA group in our study, the relevance of the observed effects on HPHC exposures in the CHTP group were interpreted in the context of previously published data. However, it is notable that Lüdicke et al.^15^ reported that many of the effects they observed with the prototype CHTP were comparable to those in the SA group.

The strengths of our study include tight control and a maximized internal validity to avoid confounding results, enabling assessment of CHTP performance when it was used exclusively.

## Conclusions

This study demonstrated that exclusive use of CHTP for 5 days resulted in significant reductions in levels of the 15 selected BoExp, ranging from 56 to 97%, compared with continued cigarette smoking. Additionally, the CHTP group had substantially decreased CYP1A2 activity, reflecting decreased formation of harmful carcinogenic metabolites usually found in cigarette smoke, and decreased mutagenic activity in the urine, based on the Ames test.

These results suggest that CHTP could be an acceptable substitute nicotine delivery product after a short period of adaptation for established smokers, who would otherwise continue to smoke, while substantially decreasing exposure to HPHCs.

## Supplementary information

Supplementary Informations.

## References

[1] Benowitz NL (2014). Emerging nicotine delivery products. Implications for public health. Ann. Am. Thorac. Soc..

[2] Abrams DB (2018). Harm minimization and tobacco control: Reframing societal views of nicotine use to rapidly save lives. Annu. Rev. Public Health.

[3] Haziza C (2016). Evaluation of the tobacco heating system 2.2. Part 8: 5-day randomized reduced exposure clinical study in Poland. Regul. Toxicol. Pharmacol. RTP.

[4] Ludicke F (2017). Effects of switching to the tobacco heating system 2.2 menthol, smoking abstinence, or continued cigarette smoking on biomarkers of exposure: a randomized, controlled, open-label, multicenter study in sequential confinement and ambulatory settings (part 1). Nicotine Tob. Res..

[5] Haziza C (2016). Assessment of the reduction in levels of exposure to harmful and potentially harmful constituents in japanese subjects using a novel tobacco heating system compared with conventional cigarettes and smoking abstinence: a randomized controlled study in confinement. Regul. Toxicol. Pharmacol..

[6] Gale N (2018). Changes in biomarkers of exposure on switching from a conventional cigarette to tobacco heating products: a randomized, controlled study in healthy Japanese subjects. Nicotine Tob. Res..

[7] IOM (Institute of Medicine). *Clearing the smoke—assessing the science base for tobacco harm reduction* (Washington, DC: The National Academies Press, accessed on 06 March 2017); https://www.nap.edu/catalog/10029.html (2001). 25057541

[8] Creamer MR (2019). Tobacco product use and cessation indicators among adults—United States, 2018. Morb. Mortal. Wkly Rep..

[9] Orleans CT, Slade J (1993). Nicotine Addiction: Principles and Management.

[10] Pederson LL, Nelson DE (2007). Literature review and summary of perceptions, attitudes, beliefs, and marketing of potentially reduced exposure products: communication implications. Nicotine Tob. Res..

[11] Felter, J. L. *et al.* (Google Patents, 2004).

[12] Caputi TL (2017). Industry watch: heat-not-burn tobacco products are about to reach their boiling point. Tob. Control.

[13] FDA (Food and Drug Administration). Guidance for industry—modified risk tobacco product applications—draft guidance (2012).

[14] Phillips BW (2018). A 90-day oecd tg 413 rat inhalation study with systems toxicology endpoints demonstrates reduced exposure effects of the aerosol from the carbon heated tobacco product version 1.2 (chtp1.2) compared with cigarette smoke. I. Inhalation exposure, clinical pathology and histopathology. Food Chem. Toxicol..

[15] Ludicke F, Haziza C, Weitkunat R, Magnette J (2016). Evaluation of biomarkers of exposure in smokers switching to a carbon-heated tobacco product: a controlled, randomized, open-label 5-day exposure study. Nicotine Tob. Res..

[16] Fagerstrom K, Russ C, Yu CR, Yunis C, Foulds J (2012). The fagerstrom test for nicotine dependence as a predictor of smoking abstinence: a pooled analysis of varenicline clinical trial data. Nicotine Tob. Res..

[17] Haziza C (2017). Biomarker of exposure level data set in smokers switching from conventional cigarettes to tobacco heating system 2.2, continuing smoking or abstaining from smoking for 5 days. Data Brief.

[18] WHO Study Group *et al.* WHO study group on tobacco product regulation—report on the scientific basis of tobacco product regulations: Fifth report of a WHO study group. (2015). 26353746

[19] FDA (Food and Drug Administration). Guidance for industry—reporting harmful and potentially harmful constituents in tobacco products and tobacco smoke under section 904(a)(3) of the federal food, drug, and cosmetic act—draft guidance (2012).

[20] Goniewicz ML (2009). Elimination kinetics of the tobacco-specific biomarker and lung carcinogen 4-(methylnitrosamino)-1-(3-pyridyl)-1-butanol. Cancer Epidemiol. Biomark. Prev..

[21] Faber MS, Fuhr U (2004). Time response of cytochrome p450 1a2 activity on cessation of heavy smoking. Clin. Pharmacol. Ther..

[22] Tricker AR (2014). Biomarkers derived from nicotine and its metabolites: a review. Beiträge zur Tabakforschung/Contrib. Tob. Res..

[23] Lindner D, Smith S, Leroy CM, Tricker AR (2011). Comparison of exposure to selected cigarette smoke constituents in adult smokers and nonsmokers in a European, multicenter, observational study. Cancer Epidemiol. Biomark. Prev..

[24] Theophilus EH, Coggins CRE, Chen P, Schmidt ER, Borgerding MF (2015). Magnitudes of biomarker reductions in response to controlled reductions in cigarettes smoked per day: a one-week clinical confinement study. Regul. Toxicol. Pharmacol..

[25] Scherer G, Urban M, Engl J, Hagedorn HW, Riedel K (2006). Influence of smoking charcoal filter tipped cigarettes on various biomarkers of exposure. Inhalation Toxicol..

[26] WHO (World Health Organization). WHO guidelines for indoor air quality: selected pollutants (2010). 23741784

[27] Mahoney JJ, Vreman HJ, Stevenson DK, Van Kessel AL (1993). Measurement of carboxyhemoglobin and total hemoglobin by five specialized spectrophotometers (co-oximeters) in comparison with reference methods. Clin. Chem..

[28] Carmella SG (2012). Correction to effects of smoking cessation on eight urinary tobacco carcinogen and toxicant biomarkers. Chem. Res. Toxicol..

[29] Park SL (2015). Mercapturic acids derived from the toxicants acrolein and crotonaldehyde in the urine of cigarette smokers from five ethnic groups with differing risks for lung cancer. PLoS ONE.

[30] Torikai K, Yoshida S, Takahashi H (2004). Effects of temperature, atmosphere and PH on the generation of smoke compounds during tobacco pyrolysis. Food Chem. Toxicol..

[31] Schettgen T, Ochsmann E, Alt A, Kraus T (2010). A biomarker approach to estimate the daily intake of benzene in non-smoking and smoking individuals in Germany. J. Expo. Sci. Environ. Epidemiol..

[32] Carmella, S. G. *et al.* Effects of smoking cessation on eight urinary tobacco carcinogen and toxicant biomarkers *Chem. Res. Toxicol.* **22**, 734–741. Erratum in Chem Res Toxicol. 2012;2025(2013):2763, http://doi.org/10.1021/tx800479s (2009). 10.1021/tx800479s PMC2704054 19317515

[33] Feng S (2006). Evaluation of urinary 1-hydroxypyrene, s-phenylmercapturic acid, trans, trans-muconic acid, 3-methyladenine, 3-ethyladenine, 8-hydroxy-2'-deoxyguanosine and thioethers as biomarkers of exposure to cigarette smoke. Biomarkers.

[34] Toxicology Data Network. 4-(N-nitrosomethylamino)-1-(3-pyridyl)-1-butanone (casrn: 64091-91-4). (accessed 20 August 2014); https://toxnet.nlm.nih.gov/cgi-bin/sis/search2/f?./temp/~bLaWqY:3 (2010).

[35] Fischer S, Spiegelhalder B, Eisenbarth J, Preussmann R (1990). Investigations on the origin of tobacco-specific nitrosamines in mainstream smoke of cigarettes. Carcinogenesis.

[36] Moldoveanu SC, Borgerding M (2008). Formation of tobacco specific nitrosamines in mainstream cigarette smoke; part 1, FTC smoking. Contrib. Tob. Res..

[37] Hecht SS (1999). Quantitation of urinary metabolites of a tobacco-specific lung carcinogen after smoking cessation. Can. Res..

[38] Mure K, Hayatsu H, Takeuchi T, Takeshita T, Morimoto K (1997). Heavy cigarette smokers show higher mutagenicity in urine. Mutat. Res..

[39] Hammond D, Fong GT, Cummings KM, Hyland A (2005). Smoking topography, brand switching, and nicotine delivery: results from an in vivo study. Cancer Epidemiol. Biomark. Preven..

[40] Hajek P (2014). Nicotine intake from electronic cigarettes on initial use and after 4 weeks of regular use. Nicotine Tob. Res..

[41] Reilly SM (2017). Effects of topography-related puff parameters on carbonyl delivery in mainstream cigarette smoke. Chem. Res. Toxicol..

[42] Baker R (1975). Temperature variation within a cigarette combustion coal during the smoking cycle. High Temp. Sci..

[43] Miyake T, Shibamoto T (1995). Quantitative analysis by gas chromatography of volatile carbonyl compounds in cigarette smoke. J. Chromatogr. A.

[44] U.S. Department of Health and Human Services. The health consequences of smoking—50 years of progress: a report of the surgeon general (2014).

[45] Sitkauskiene B, Stravinskaite K, Sakalauskas R, Dicpinigaitis PV (2007). Changes in cough reflex sensitivity after cessation and resumption of cigarette smoking. Pulm. Pharmacol. Ther..

[46] Dicpinigaitis PV (2006). Effect of smoking cessation on cough reflex sensitivity. Eur. Respir. J..

[47] Etter JF (2010). Short-term change in self-reported copd symptoms after smoking cessation in an internet sample. Eur. Respir. J..

[48] Gratziou C (2009). Respiratory, cardiovascular and other physiological consequences of smoking cessation. Curr. Med. Res. Opin..

[49] Hughes JR (2007). Effects of abstinence from tobacco: valid symptoms and time course. Nicotine Tob. Res..

[50] Sitkauskiene B, Dicpinigaitis PV (2010). Effect of smoking on cough reflex sensitivity in humans. Lung.

[51] Motooka Y (2018). Adverse events of smoking cessation treatments (nicotine replacement therapy and non-nicotine prescription medication) and electronic cigarettes in the food and drug administration adverse event reporting system, 2004–2016. SAGE Open Med..

