# Supplementary material for: Reduced levels of biomarkers of exposure in smokers switching to the Carbon-Heated Tobacco Product 1.0: a controlled, randomized, open-label 5-day exposure trial
Source: Sci Rep. 2020 Nov 5;10:19227. doi: 10.1038/s41598-020-76222-y (PMC7644773; doi:10.1038/s41598-020-76222-y)

Reduced Levels of Biomarkers of Exposure in Smokers Switching to the Carbon-Heated Tobacco Product 1.0: A Controlled, Randomized, Open-Label Five-Day Exposure Trial

Cam Tuan Tran, MD; Marija Bosilkovska, PhD; Guillaume de La Bourdonnaye; Nicolas Blanc, MD; Christelle Haziza, PhD

Supplementary Material

Supplementary Table 1. Biomarkers of Exposure to Selected HPHCs.

Acronym	Biomarker of exposure	HPHC	Toxicity class
COHb	Carboxyhemoglobin [1]	Carbon monoxide	RDT
3-HPMA	3-hydroxypropylmercapturic acid	Acrolein	RT, CT
MHBMA	Monohydroxybutenylmercapturic acid	1,3-Butadiene	CA, RT, RDT
S-PMA	S-phenylmercapturic acid	Benzene	CA, CT, RDT
Total 1-OHP[2]	Total 1-hydroxypyrene	Pyrene	CA
4-ABP	4-aminobiphenyl	4-aminobiphenyl	CA
1-NA	1-aminonaphthalene	1-aminonaphthalene	CA
2-NA	2-aminonaphthalene	2-aminonaphthalene	CA
o-tol	o-toluidine	o-toluidine	CA
CEMA	2-cyanoethylmercapturic acid	Acrylonitrile	CA, RT
HEMA	2-hydroxyethylmercapturic acid	Ethylene oxide	CA, RT, RDT
3-HMPMA	3-hydroxy-1-methylpropylmercapturic acid	Crotonaldehyde	CA
Total 3-OH-B[a]P	Total 3-hydroxy-1-methylpropyl-mercapturic acid	Benzo[a]pyrene	CA
Total NNAL[3]	Total 4-(methylnitrosamino)-1-(3-pyridyl)-1-butanol	4-(methylnitrosamino)-1-(3-pyridyl)-1-butanone (NNK)	CA
Total NNN	Total N-nitrosonornicotine	N-nitrosonornicotine (NNN)	CA

List of HPHCs and acronyms used. Biomarker matrix is 24-hour urine if not stated otherwise. 1) Matrix for COHb was blood. 2) Total 1-OHP was determined as the molar sum of 1-hydroxypyrene and its glucuronide and sulfate conjugates. 3) Total NNAL was determined as the molar sum of 4-(methylnitrosamino)-1-(3-pyridy1)-1-butanol and its O-glucuronide conjugate. Abbreviations: HPHC, harmful or potentially harmful constituent; CA, carcinogen, RT, respiratory toxicant; RDT, reproductive and development toxicant; CT, cardiovascular toxicant.

Supplementary Table 2. Levels of Reductions of Biomarkers of Exposure on Day 5, CHTP Relative to Cigarettes. % Reduction Defined as (1-CHTP/cigarettes)%

Biomarkers	% reduction (95% CI)
COHb (%)	58.83 (49.30, 66.56)
3-HPMA (ng/mg creatinine)	63.53 (59.55, 67.12)
MHBMA (pg/mg creatinine)	82.82 (78.79, 86.09)
S-PMA (pg/mg creatinine)	88.14 (86.46, 89.62)
Total 1-OHP (pg/mg creatinine)	55.57 (51.39, 59.39)
4-ABP (pg/mg creatinine)	78.87 (76.24, 81.21)
1-NA (pg/mg creatinine)	97.09 (96.50, 97.59)
2-NA (pg/mg creatinine)	90.05 (88.65, 91.28)
o-tol (pg/mg creatinine)	72.08 (68.60, 75.18)
CEMA (ng/mg creatinine)	85.83 (84.02, 87.44)
HEMA (pg/mg creatinine)	65.11 (59.54, 69.92)
3- HMPMA (ng/mg creatinine)	75.52 (72.56, 78.17)
Total 3-OH-B[a]P (fg/mg creatinine)	77.12 (72.35, 81.07)
Total NNAL (pg/mg creatinine)	57.74 (53.00, 62.00)
Total NNN (pg/mg creatinine)	70.19 (62.17, 76.51)

Biomarkers of exposure including COHb measured in blood and those in 24-hour urine creatinine-adjusted samples. Values are geometric least square mean reductions and 95% CIs of log-transformed Day 5 biomarker values from an ANCOVA model, with study arm, sex, and cigarette consumption reported at screening and baseline biomarker values as fixed-effect factors. Biomarker of exposure levels at baseline and Day 5 as well as percent change are presented separately for the CHTP 1.0 and cigarette groups in Table 2. Acronyms of biomarkers of exposure are described in Supplementary Table 1. Abbreviation: CI, confidence interval.

Supplementary Table 3. Daily Product Use by Study Group.

Time point	CHTP (n = 41)<br>Mean ± SD (range)	Cigarettes (n = 39)<br>Mean ± SD (range)
Baseline Cigarettes	17.8 ± 3.6 (9–26)	17.1 ± 2.6 (10–24)
Day 1	16.8 ± 4.6 (10–29)	15.1 ± 2.8 (11–21)
Day 2	21.6 ± 6.1 (12–41)	16.5 ± 3.4 (9–27)
Day 3	21.6 ± 6.0 (13–40)	16.6 ± 3.4 (10–26)
Day 4	19.9 ± 6.3 (10–41)	16.4 ± 3.2 (11–25)
Day 5	25.8 ± 8.3 (10–56)	20.3 ± 3.8 (12–28)

Abbreviations: CHTP, Carbon-Heated Tobacco Product 1.0; Mean, arithmetic mean; SD, standard deviation.

Supplementary Table 4. Nicotine Uptake Levels.

Biomarker	CHTP (n = 41)<br>Geometric mean (95% CI)	Cigarettes (n = 39)<br>Geometric mean (95% CI)
Neq (mg/g creatinine) (urine)		
Baseline	9.20 (8.12; 10.43)	8.78 (7.63; 10.10)
Day 5	11.56 (9.60; 13.93)	10.82 (9.47; 12.35)
Nicotine (ng/mL) (plasma)		
Baseline	13.39 (11.71; 15.32)	12.96 (11.34; 14.80)
Day 5	15.60 (13.03; 18.69)	15.80 (13.93; 17.93)
Cotinine (ng/mL) (plasma)		
Baseline	212.05 (190.48; 236.07)	204.38 (182.72; 228.60)
Day 5	259.30 (222.59; 302.07)	244.48 (219.00; 272.93)

Abbreviations: CI, confidence interval; CHTP, Carbon-Heated Tobacco Product 1.0; Neq, nicotine equivalents (the molar sum of urinary nicotine, cotinine, and trans-3’-hydroxycotinine plus their respective glucuronide conjugates).

Supplementary Table 5. Human Puffing Topography Parameter Values at Baseline, Day 1, and Day 4 as well as % Excess of CHTP Relative to Cigarettes.

Parameter<br>Time	n	CHTP<br>Geometric Mean<br>(CV%)	n	Cigarettes<br>Geometric Mean<br>(CV%)	% CHTP/Cigarettes –100<br>(95% CI)
Total puff volume (mL)					
Baseline	25	721.7 (23.45)	22	762.4 (21.64)	
Day 1	38	916.5 (48.68)	21	726.1 (22.98)	38.19 (16.69; 63.65)
Day 4	39	1198.8 (42.82)	21	731.2 (24.61)	69.77 (43.17; 101.31)
Average puff volume (mL)					
Baseline	25	52.86 (31.06)	22	53.61 (22.54)	
Day 1	38	49.37 (46.74)	21	51.89 (15.81)	1.84 (–11.30; 16.93)
Day 4	39	57.50 (42.07)	21	51.83 (15.99)	12.73 (–1.63; 29.17)
Average puff duration (s)					
Baseline	25	1.83 (40.33)	22	1.56 (27.89)	
Day 1	38	1.99 (49.03)	21	1.49 (24.06)	24.56 (11.12; 39.62)
Day 4	39	2.29 (50.58)	21	1.49 (21.86)	40.72 (21.72; 62.69)
Total puff duration (s)					
Baseline	25	24.94 (40.34)	22	22.25 (29.30)	
Day 1	38	36.86 (51.23)	21	20.90 (31.68)	64.82 (44.24; 88.33)
Day 4	39	47.69 (52.26)	21	20.99 (30.22)	105.60 (76.21; 139.89)
Total number of puffs					
Baseline	25	13.7 (25.33)	22	14.3 (22.48)	
Day 1	38	18.6 (22.72)	21	14.0 (24.56)	33.73 (24.18; 44.00)
Day 4	39	20.9 (29.51)	21	14.1 (27.30)	49.14 (31.56; 69.07)
Puff frequency (puffs/min)					
Baseline	25	3.89 (31.72)	22	4.03 (23.85)	
Day 1	38	4.81 (24.59)	21	4.12 (27.57)	20.36 (8.78; 33.17)
Day 4	39	5.16 (33.75)	21	4.26 (28.16)	26.08 (10.54; 43.80)

Values are per stick. CHTP/cigarette ratios are based on adjusted geometric least square means and 95% confidence intervals from an ANCOVA model conducted on log-transformed values, with log-transformed baseline value, study group, sex, and cigarette consumption reported at screening as fixed-effect factors. Abbreviations: Mean, arithmetic mean; SD, standard deviation; CHTP, Carbon-Heated Tobacco Product 1.0.

Supplementary Table 6. Adverse Events.

Adverse events	CHTP<br>n = 41<br>n (%)	Cigarettes<br>n = 39<br>n (%)	Discontinued before randomization<br>n = 5<br>n (%)	Overall<br>n = 85<br>n (%)
Subjects with AEs (%)	31 (75.6)	20 (51.3)	5 (100)	56 (65.9)
AEs (occurring in >4% of all subjects)				
Headache	19 (46.3)	9 (23.1)	3 (60)	31 (36.5)
Syncope	2 (4.9)	1 (2.6)	1 (20)	4 (4.7)
Cough	13 (31.7)	0	0	13 (15.3)
Constipation	2 (4.9)	1 (2.6)	0	3 (3.5)
Puncture site hematoma	0	2 (5.1)	0	2 (2.4)
Hypertriglyceridemia	0	2 (5.1)	2 (40)	4 (4.7)
Leukocytosis	2 (4.9)	0	1 (20)	3 (3.5)
Lymphocytosis	2 (4.9)	0	0	2 (2.4)
Hyperbilirubinemia	0	2 (5.1)	1 (20)	3 (3.5)

Percentages calculated on n of subjects (column headers). Abbreviations: AE, adverse event; CHTP, Carbon-Heated Tobacco Product 1.0.

Supplementary Figure 1. A Complete CHTP 1.0 Tobacco Stick

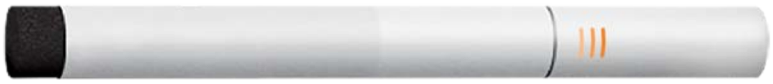

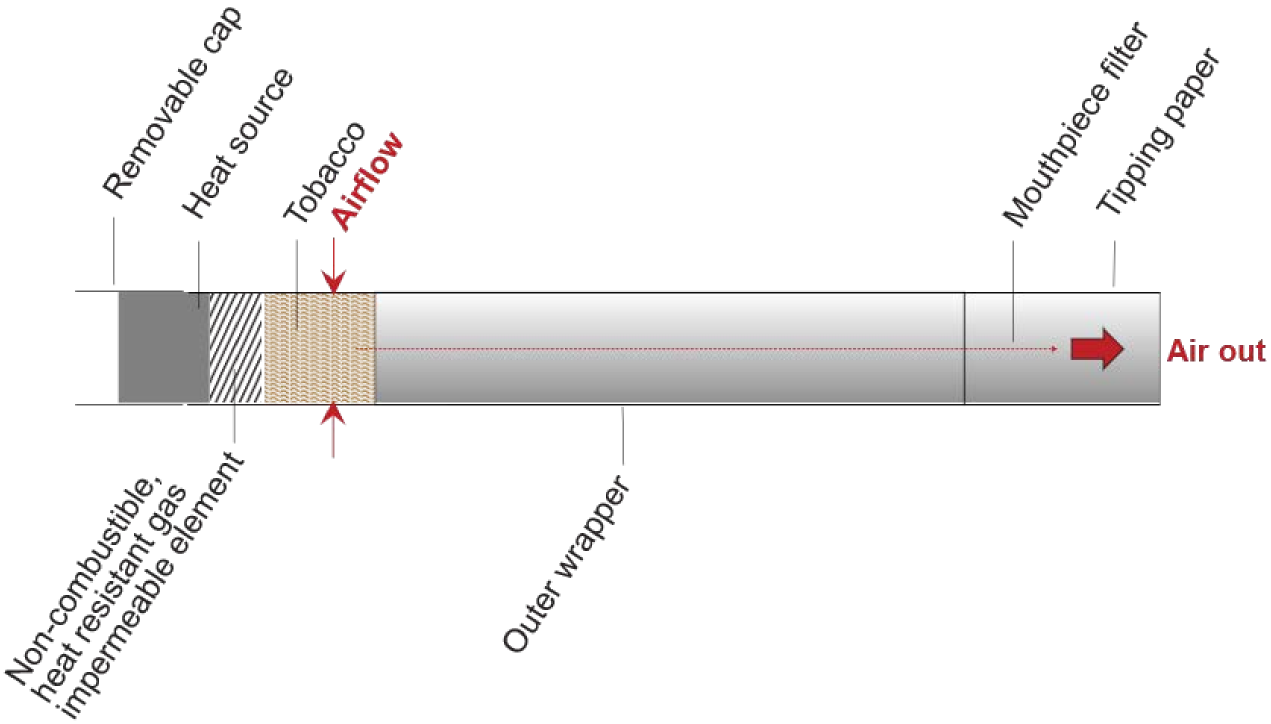

Supplement: Supplementary file 1 — Supplementary Informations. [file 41598_2020_76222_MOESM1_ESM.pdf]
